# Building Bridges for “Palliative Care-in-Place”: Development of a mHealth Intervention for Informal Home Care

**DOI:** 10.3389/fpsyg.2022.862347

**Published:** 2022-03-25

**Authors:** Carlos Laranjeira, Maria Anjos Dixe, Ricardo Martinho, Rui Rijo, Ana Querido

**Affiliations:** ^1^School of Health Sciences of Polytechnic of Leiria, Leiria, Portugal; ^2^Centre for Innovative Care and Health Technology (ciTechCare), Polytechnic of Leiria, Leiria, Portugal; ^3^Research in Education and Community Intervention (RECI I&D), Piaget Institute, Viseu, Portugal; ^4^Center for Health Technology and Services Research (CINTESIS), University of Porto, Porto, Portugal; ^5^Technology and Management School of Polytechnic of Leiria, Leiria, Portugal

**Keywords:** selfcare, chronic disease, mHealth, technology, palliative care, caregivers, communication

## Abstract

**Background:**

In Palliative Care (PC), family and close people are an essential part of provision of care. They assume highly complex tasks for which they are not prepared, with considerable physical, psychological, social and economic impact. Informal Caregivers (ICs) often falter in the final stage of life and develop distress, enhancing emotional burden and complicated grief. The lack of available and accessible in-person counselling resources is often reported by ICs. Online resources can promote early access to help and support for patient-IC dyads in palliative care. The primary aim of this research is to co-design, develop and test the feasibility of the Help2Care-PAL mHealth app that addresses the needs of ICs of palliative patients cared for at home. This Digital Health Intervention (DHI) in palliative care will be used for education, symptom management, communication and decision-making, to enhance Quality of Life (QoL) of patients and ICs, fostering anticipatory grief and the reach and efficiency of services.

**Methods:**

This study will use an iterative co-design process and convergent mixed-methods design, following the MORECare consensus for developing a complex intervention. Construction of the DHI will follow four main phases: (I) a needs assessment (a cross-sectional survey, individual interviews with ICs and focus groups with professionals with community palliative care experience); (II) design and co-production of mHealth materials and interventions to support ICs; (III) the development of a mHealth app; and (IV) usability and feasibility of the mHealth app. The Help2Care-PAL platform seeks to build resources from the perspectives and needs of both family dyads and nursing professionals working in the field of community palliative care. User-centeredness will be ensured by the active participation of patient-IC dyads and professionals of the palliative care community.

**Discussion:**

This mixed-method study will offer new insights on needs and expectations of patient-IC dyads and nurses in community palliative care regarding caregiving preparedness and online health resources. Through the implementation of an adaptive digital tool, we aim to improve access to palliative care family support, which is highly linked with the wellbeing of patients and especially new ICs.

## Introduction

Community Palliative Care Services provide access to expert support by Healthcare Professionals (HCPs) for patients facing a life-threatening condition, irrespective of diagnosis, in all community care settings. The service teams comprise professionals with Specialist Palliative Care Competencies (National Hospice and Palliative Care Organization, 2021). In addition to clinical caseload, within agreed boundaries and protocols, these teams provide education and therapy, and perform consultancy services and research. Their strategies include ‘symptom management, medication management, family support and training, advance care planning and goals of care facilitation, caregiver respite, and interventions for emotional and spiritual needs of patients and family’ (Portz et al., 2020, p. 22).

The United Nations Sustainable Development 2030 Agenda (United Nations, 2015) set the goals of ‘modern and effective care for all [and] conditions for all people to attain their fundamental rights to health and well-being’ (Widberg et al., 2020, p. 2). Moreover, improving access to and quality of palliative care delivery is a healthcare priority in many countries. Information technologies, in particular, can help promote access to health information and care, particularly palliative care (Finucane et al., 2021). For instance, telehealth is an alternative form of palliative care delivery, which allows patients to spend more time or even remain at home, if they wish, throughout their illness (Steindal et al., 2021). However, Digital Health Interventions (DHIs) in palliative care must consider the patient’s overall needs and preferences, while fostering a relationship that promotes dignity and is focused on the patient and caregiver’s values and goals (Widberg et al., 2020).

Digital health, or eHealth, refers to applications of Information and Communication Technologies (ICTs) to all aspects of patient care and health services, including patient self-management, and encompasses a range of related concepts, such as ‘telemedicine and telehealth, mobile health (mHealth), health informatics, and wearable devices’ (Finucane et al., 2021, p. 1; Steindal et al., 2021). Digital resources are already part of routine healthcare practice in many countries, from simply maintaining electronic health records and using decision support software to using videoconferencing to remotely interact with patients. Mobile phones, apps, wearables and social media are already widely used by citizens/patients, and new technologies—such as ‘augmented reality, virtual assistants, and artificial intelligence’—are increasingly applied in clinical management and patient self-care. As they become more affordable and widespread, these technologies are reshaping healthcare (Finucane et al., 2021, p. 1).

Palliative care is one area where these technologies are increasingly being deployed. Various eHealth applications enable patients to participate in and govern their own care, for example by self-reporting symptoms and needs (Cooley et al., 2017; Guo et al., 2017; Pinto et al., 2017; Vitacca et al., 2019; Finucane et al., 2021). The possibility of sending text messages to HCPs, for example, to give notice that medication needed refilling, is perceived by patients as a well-functioning alternative for traditional oral and synchronous communication. Using validated instruments, patients can provide HCPs with information, using eHealth technology, on physical, mental, social and existential symptoms, as well as Quality of Life (QoL), which can then be used to make care and treatment decisions (Hennemann-Krause et al., 2015; Tieman et al., 2016; Cooley et al., 2017; Pinto et al., 2017; Bonsignore et al., 2018; Vitacca et al., 2019). By enabling informal home care, eHealth technology can help ICs maintain their social relationships and contacts during their daily life. Participation in social contexts, in caring and supportive situations, despite troublesome symptoms and severe illness, provides a positive connection to life and loved ones. Furthermore, by enhancing the skills and psychological wellbeing of ICs, these technologies contribute towards improving the QoL, digital literacy and social inclusion of the care receiver (Widberg et al., 2020; Gomes et al., 2021).

As in other European countries, care for palliative patients in Portugal relies on ICs. However, due to financial hardships aggravated by the COVID-19 sanitary crisis, most family carers cannot provide care for their relatives (Parmar et al., 2021). Families often rely on the help of commodified care, benefiting from solutions proposed by social security services (Brito, 2019). There has also been an expansion of the private sector in end-of-life care. While this means more people might have access to palliative care, it also implies increased inequality (Gomes et al., 2020). To respect IC preferences for providing care at home, it is necessary to develop quality home-based palliative care services, focusing on training and the expansion of field teams. Despite this preference, home care for patients with palliative needs increases caregiving burden which usually increases as the patient’s condition declines (Guerriere et al., 2016; Ahn et al., 2020). An estimated 10–60% of caregivers experience negative psychological and physical consequences, including anxiety, depression, grief and poor physical health (Ahn et al., 2020). These results highlight the importance of care for caregivers wishing to support death at home.

The rising need for palliative care services in Portugal, combined with the increasing use of DHIs in healthcare, calls for studies on how to adequately design such online resources. Although mHealth palliative care apps are available, these early apps did not offer family caregiving tools (Portz et al., 2020). Disease-specific apps are often too limited, as palliative care patients often have more than one condition. Moreover, current mHealth apps for palliative care do not offer comprehensive tools for coping with bereavement and grief, proving most useful early in the bereavement process (i.e., soon after the death of a loved one; Portz et al., 2020). While grief occurs throughout the course of serious illness (anticipatory grief; Nielsen et al., 2016), most apps are tailored for coping with bereavement after patient death. A recent review indicates that there are few commercial apps specifically for caregiving (Portz et al., 2020) and little usability evidence on caregiver apps (Grossman et al., 2018; Quinn et al., 2019).

Therefore, this study will generate a DHI that can be used by caregivers of palliative patients living in remote communities of Portugal. Despite long distances, patients could receive care through eHealth applications, in an accessible manner, without spending time or money on travel (Melton et al., 2017; Widberg et al., 2020). The Help2Care-PAL’s purpose is to boost and sustain the role of ICs in making the ‘palliative care-in-place’ imperative a sustainable reality in rural and remote areas. Furthermore, and compared with other digital solutions, this DHI will enable tailored care (specific information) according to patient and family needs, monitored and prepared by a health professional. All the information presented to the caregiver is validated by a professional, guaranteeing the carer does not get the wrong information (Gomes et al., 2021).

### Theoretical Framework

The guidelines from the National Consensus Project (NCP) for Quality Palliative Care offer an opportunity to reassess the domains of care delivered at home, such as physical, psychological, social or spiritual aspects of care. Anderson et al. (2018) attributes particular importance to the family/caregiver domain. In this study, families described their role as care managers, but also reflected upon their families and personal transformations resulting from caregiving. The same authors considered two additional domains—financial/legal and legacy/bereavement—that reflect concerns about the preservation of assets and life transitions, respectively.

In addition, Social Convoy Theory (Antonucci et al., 2014) conceptualises social relations as a convoy that includes informal support from family members, friends and neighbours and formal supports such as professional caregivers. This model provides a framework for the complex relationships between individuals that give and receive social support. Social convoys have been shown to improve health outcomes and QoL among patients with serious illness, and reduce mortality in older populations (Portz et al., 2020). Palliative care should also follow a team-based approach with continuous reciprocal information flow between patients and care providers, rather than exclusive dissemination from one party (Portz et al., 2020).

Despite the evidence indicating social support as a critical construct in improving health behaviours and health outcomes, mHealth platforms are designed for individual users and usually do not integrate the patient’s family, friends and professional support to maximise benefit (Portz et al., 2020).

### Aim and Research Questions

The primary aim of the study is to co-design, develop and test the feasibility of a novel mHealth app that addresses the needs of ICs of palliative patients who are cared for at home. This study aims to contribute towards preparedness for end-of-life care and reducing potential negative effects for ICs. The following specific research questions will be addressed as:

- What is the preparedness of ICs when facing the care of people with palliative needs?
- How can a mHealth app be tailored to address the needs of carers regarding palliative care management?
- How can a mHealth app be used effectively by ICs to learn and receive support in their caregiving role?

## Design

This study will use an iterative co-design process and convergent mixed-methods design, which ‘involves simultaneously collecting and analysing qualitative and quantitative data and, as critically important, taking into consideration the iterative nature of mHealth technology development’ (Alwashmi et al., 2019, p. 5). Additionally, we will follow the MOREC are consensus for developing a complex intervention in PC (Farquhar et al., 2013). Complex interactions between the intervention and its context determine and shape whether and how outcomes are generated (Skivington et al., 2021). Based on prior work of Rathnayake et al. (2018), this flexible approach will promote a comprehensive understanding of the phenomenon of interest, matching the design and evaluation of a multi-phase complex intervention.

### mHealth Development

The DHI will be constructed in four main phases, implemented between April 2022 and December 2022, in Portugal. The research team has experience in both qualitative and quantitative research and holds advanced degrees in nursing (CL, AQ and MD) and software engineering (RM and RR).

**Figure 1:**
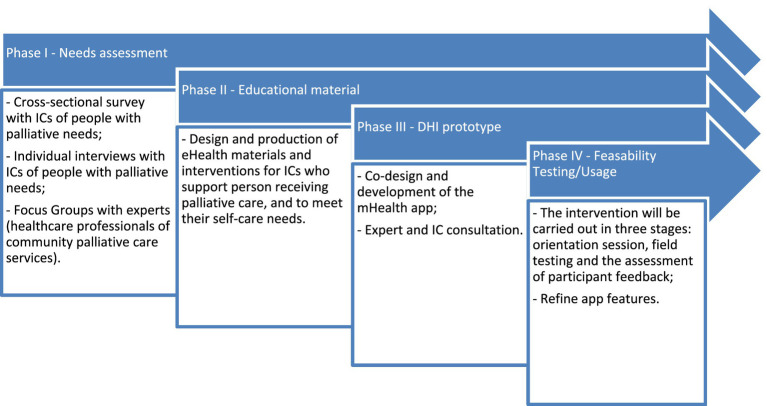
mHealth app development process.

The four phases are (I) a needs assessment; (II) design and co-production of mHealth materials and interventions to support ICs; (III) the development of a mHealth app; and (IV) usability and feasibility of the mHealth app (see Figure 1).

#### Phase I (Needs Assessment)

Phase I (needs assessment) will involve the identification of the target audience’s needs through a cross-sectional survey, individual interviews with ICs and focus groups with professional experts with community palliative care experience. As much as possible, this phase will integrate the attitudes and knowledge of all potential users in the development of training materials and the design of the DHI.

- The cross-sectional survey (including online or phone-based questionnaire) will assess the needs of ICs in their caregiving functions and the use of mHealth apps in health-seeking behaviours (Rathnayake et al., 2018). The participating ICs living in the community will be adults, unpaid primary carers, that provide informal care at home. Sample recruitment will be based on nationally representative data, based on the service list of the community support teams in palliative care from the Portuguese Observatory of Palliative Care. The surveys will draw from a random stratified sample of ICs based on gender, age range and geographical location. According to the Portuguese Strategic Plan for the Development of Palliative Care (2019-2020 biennium; Diário da República Portuguesa, 2019), there are an estimated 90,000 Portuguese people in need of palliative care, and 54 community support teams in palliative care with a regional coverage. To obtain enough data with a margin error of 5%, and a confidence level of 95%, we plan to recruit at least 400 ICs. A structured questionnaire divided into five sections will be used for data collection. The section “Introduction” will collect sociodemographic information of the caregiver and health condition of the care recipient, including the (i) Edmonton Symptom Assessment Scale (Chang et al., 2000) to rate the intensity of common symptoms experienced by palliative patients and the (ii) Performance Palliative Scale (PPS) to estimate the survival time of palliative patients (Victoria Hospice Society, 2016). In section “Design”, the health literacy level of carers will be assessed with three self-reported questions proposed by Chew et al. (2004), which show high specificity for detecting inadequate health literacy (Chew et al., 2004). Questions are answered using a five-point Likert scale (never-1 to always-5). Higher scores indicate a lower level of health literacy (Levin et al., 2014). In section “Ethical Considerations”, the needs of the patient’s family will access with Family Inventory of Needs (Kristjanson et al., 1995) validated in Portugal by Areia et al. (2016a). This instrument has 40 items and quantifies two concepts: the importance of family care needs and fulfilment of care needs. Regarding to internal consistency, a high Cronbach’s alpha coefficient was obtained for the Importance of Needs subscale (*α* = 0.89) and for the Needs Satisfaction subscale (*α* = 0.91). Section “Dissemination Strategies” will assess the Caregiver Burden Scale, which was based on Zarit’s Burden Interview Scale, and has been translated and validated in Portugal by Sequeira (2010). This scale assesses the objective and subjective burden of informal care and collects information on health, social life, personal life, financial situation, emotional situation and type of relationship. The instrument contains 22 items rated on a 5-point Likert scale ranging from 1 (never) to 5 (almost always), whereby the total score ranges between 22 and 100, with higher scores indicating a greater burden. A score of 56 or more was considered a high burden. This scale boasted an internal consistency of *α* = 0.82. In section “Discussion”, the pre-death grief will be assessed by the Marwit-Meuser Caregiver Grief Inventory Short-Form (MM-CGI-SF; Marwit and Meuser, 2005), validated in Portugal by Areia et al. (2016b). This form has 18 items rated on a 5-point Likert scale, thus a maximum score of 90. Before patient death, caregiver responses to perceived losses may include anticipation of future loss following physical death and mourning of present loss due to psychological death (Liew et al., 2018). The MM-CGI-SF had a good internal consistency (*α* = 0.89).
- Qualitative data collection using individual in-depth interviews with ICs of people with palliative needs (in-person or telephone/videoconference interviews) will explore perceived preparedness for different domains of caregiving, including communication with relatives and considerations about the IC’s future, including bio-psycho-social-cultural and spiritual issues. Educational needs may include medical issues, such as symptoms and symptom relief; planning for the moment of death; and tracking IC’s burden and risk of complicated grief. Interviews will investigate IC perspectives and challenges when caring at home and using mHealth apps in health-seeking behaviours (Rathnayake et al., 2021). Three broad questions will be used to stimulate conversation, ‘Please, describe your preparedness to provide informal palliative home care’; ‘What are the activities you find more difficult to perform for your care recipient?’; and ‘Do you think a smartphone app would be helpful for you to manage the daily activities?’, followed by further follow-up questions, depending on answers provided previously. Sociodemographic variables such as age, gender and education level will also be collected. Overall information will be used to design the content and features of the proposed mHealth app. Participants from the cross-sectional survey who consent will be invited to participate in a qualitative interview. A purposive, maximum-variation sampling technique will be adopted to recruit 12 ICs. Each interview session will last for about 40–60 min and will be recorded digitally.
- Qualitative data collection through virtual Focus Groups (FG) with professional experts in palliative care. FGs are suitable for eliciting attitudes, expectations and emotional arguments during the interactive group process (Richard et al., 2021). In line with the study of Rathnayake et al. (2018), the semi-structured interviews will collect expert opinions regarding the provision of palliative care (especially management of care) and about mHealth app for carers, including knowledge of existing smartphone-based interventions, attitudes towards mHealth educational interventions and possible constraints and challenges towards the development of a mHealth app. The interview guide will include some open-ended questions (e.g., ‘What are the main difficulties you face when managing/planning daily activities for people with palliative needs?’, ‘What are your perceptions about mHealth apps as an educational and supportive resource for ICs?’, ‘What do you think are the most important smartphone features (for examples text, video, alert functions, calendar scheduling etc.) that should be included in a mHealth app for daily living activities?’ and ‘What are the potential difficulties and concerns associated with the development of a mHealth app for ICs?’). A convenience sampling method will be used to recruit 20 experts, including HCPs (e.g., nurses, therapists, social workers, physicians and psychologists) who work in a community support team and have three or more years of experience in palliative care. Two independent online FG sessions will be conducted with no more than ten participants per group. Each FG will last for approximately 60 min and will be moderated by one palliative care nurse and a software engineer with experience in apps development. All FG sessions will be digitally recorded.

Descriptive and inferential statistics will be used to analyse the sample data of ICs and of experts who participate in survey and interviews. All statistical analyses will be conducted using SPSS v.28 (IBM). In qualitative data analysis, audio files will be transcribed verbatim. Transcripts will be analysed using WebQDA^®^ software. Thematic analysis will be conducted to identify the conceptual map of the core themes resulting from the interviews (Braun and Clarke, 2006). Themes will be inferred inductively by one researcher and discussed with the research team.

#### Phase II (Educational Material)

Phase II (educational material) includes the design and production of eHealth materials and interventions to support ICs. Data from phase I will inform the content of these materials and interventions. In this phase, an iterative co-design process (Rathnayake et al., 2018, 2021) will generate the educational material in the form of images, texts and audio/video that allow the IC to respond to the care of their family member and meet their self-care needs. The educational material, in Portuguese, will be presented at the Help2Care-PAL website, through videos and informative texts. To increase caregiving preparedness, the videos will show conversations, between ICs (played by actors) and different professionals (authentic), addressing intervention topics. The informative texts will deepen and broaden the topics raised in the videos. As a part of the intervention, the website will also host an online peer-support discussion forum, which can be used by ICs to communicate with others in a similar situation (Alvariza et al., 2020).

#### Phase III (DHI Prototype)

Phase III (DHI prototype) comprises the co-design and development of the mHealth app (Help2Care-PAL) to provide access to appropriate training materials, evaluate caregiver knowledge about these materials and collect feedback about their use, so that materials can be reviewed when necessary, offering a greater learning capacity and improved caring skills, especially for new ICs (Gomes et al., 2021). The health literacy concepts proposed by Broderick et al. (2014 cited in Rathnayake et al., 2018)—such as ‘write actionable content’, ‘display content clearly’, ‘organize and simplify’ and ‘engage users’—will be incorporated. The web application will help manage users (administrators, health professionals and caregivers), patient needs and training materials. Among other features, questionnaires can be used to verify whether caregivers or self-care patients can perform a certain task or use a specific material (Gomes et al., 2021). Through the mobile application, the health professional will be able to provide the caregiver with training materials, according to patient needs. Besides, ‘the caregiver will be able to communicate with the health professional, in case of doubts about their tasks’ (Gomes et al., 2021, p. 222).

According to Portz et al. (2020, p. 28), specific palliative care issues typically not addressed by apps include care roles (e.g., defining and providing education on roles), skills and coping (e.g., time management, stress reduction, or anticipatory grief), and communication (e.g., planning family meetings, conflict resolution, or assertiveness training)’. Therefore, the DHI prototype will cover five main topics: (1) holistic approach to palliative symptom management; (2) preparedness for caring during dying process; (3) dealing with anticipatory grief; (4) feeling connected and supported; (5) valuing oneself as a caregiver and an individual; and (6) maintaining control of the caring situation and coordinating care (see Table 1).

**Table 1:** The content of the mhealth application.

Holistic approach to palliative symptom management	Overview of palliative care
	Overview of the illness trajectory and palliative care
	Pharmacological and non-pharmacological measures for symptom relief
Preparedness for caring during dying process	Reflection on the “road map” of the dying process and how to accompaing the patient in his/her journey
	Practice of gratitude, forgiveness and spirituality
	Management of daily living activities (ex-feeding/nutrition; medication management; oral care; bathing; continence care; etc.)
	Dignity, last wishes fullfimlent and legacy
Dealing with anticipatory grief	Antecipatory grief symptoms
	Antecipatory grief stages
	Redefine hope and focus on quality of life
Feeling connected and supported	Peer support and carer support network
	Patient death and death rituals
	Advocate for better end-of-life care for everyone
Valuing oneself as a caregiver and an individual	Stress management
	Positive mental health strategies
	Self-care strategies
Maintaining control of the caring situation and coordinating care	Support services available
	Calendar planning and task reminders
	Connection to oneself and information sharing with the palliative care team

Expert consultation will review content for readability. In this phase, six elements will be invited as: three experts from the expert consultation and three ICs who participated in the qualitative interview. The prototype content will be assessed for appropriateness and clarity. Experts will rate module content based on a 4-point Likert scale, from strongly agree (1) to strongly disagree (4). A Content Validity Index (CVI) greater than 0.80 will be considered adequate (Polit et al., 2007). If their rating is worse than ‘agree’, they will be asked to share their ideas for improvement. After this step, the prototype will be concluded, and the software engineers will design the app. The app will be developed for both iOS and Android Operating Systems by computer science students undertaking a master’s degree in Computer Science, under supervision by an experienced software engineer.

#### Phase IV: Usability and Feasibility of mHealth Application

The feasibility study will include user testing, specifically field testing in a real-world setting to assess user experience with the app (Rathnayake et al., 2018). Unlike statistical null hypothesis testing, feasibility studies do not require large sample sizes (Tickle-Degnen, 2013; Rathnayake et al., 2018). The sample size will be eight ICs of people with palliative needs, living in the Alentejo Region (Portugal) and having Internet access. Participants will be recruited through qualitative interviews, where they provide information about their digital skills. Participants unconfident in these skills will receive training. All participants will be given access to the app and a researcher will demonstrate how to use it. Participants will record the frequency of usage, challenges and constraints faced when using the app (Rathnayake et al., 2018).

After the 4 weeks of field testing, a phone survey will be performed to receive feedback from users. Direct observation of user testing will be adopted to perform the usability tests (Nielsen, 1994). Here, users will be asked to perform a pre-defined number of tasks in the app, thus providing statistical data about time of execution, number of erroneous interactions (e.g., wrong clicks) and rate of successful completion.

The quality of the mHealth app will be assessed by the Mobile App Rating Scale (uMARS; Stoyanov et al., 2015, 2016). This 20-item scale, rated on a 5-point scale from (1-Inadequate to 5-Excellent), includes five quality subscales: engagement, functionality, aesthetics, information and subjective quality. User satisfaction with the app will also be evaluated by one self-assessment question: ‘How do you rate your satisfaction with this app?’ based on a 5-point scale from very satisfied to very dissatisfied (Rathnayake et al., 2018).

## Ethical Considerations

Ethical approval was obtained from the Unidade Local de Saúde—Baixo Alentejo and from the Polytechnic of Leiria. Participants will be assured that all information will be confidential and anonymous that participation is voluntary and may be suspended at any stage without penalty. Informed consent will be sought for each stage. Volunteers will receive no compensation for their participation. Coded information will be used in data management as a measure of privacy protection.

Data will be kept secure and confidential, according to institutional rules for data storage. The consent forms, data collection forms and verbatim transcripts will be kept for 10 years.

## Dissemination Strategies

The main results will be disseminated on relevant social media and websites and through short reports to participating entities and stakeholders. Scholarly papers will also be submitted to relevant peer-reviewed publications. Additionally, public presentations will be delivered to relevant national and international conferences.

## Discussion

eHealth applications can provide palliative patient-IC dyads with access to convenient information and support contacts, thus empowering them to participate and govern their self-care, and enhancing their sense of security. ‘At organizational and societal levels, eHealth may contribute to sustainable development and more efficient use of resources in palliative care’ (Widberg et al., 2020, p. 12). eHealth should complement face-to-face meetings and not replace physical meetings. ‘Human contact and human interactions are inherent to nursing, and relationships between patients and HCPs are important in palliative care’. Nonetheless, the tentative positive experiences of eHealth applications should not be discarded, namely, in our present uncertain time (Widberg et al., 2020, p. 11).

This proposed DHI will guide the development and testing of an educational and capacitating mHealth app for ICs of people with palliative needs, and generate knowledge contributing to health improvement (Skivington et al., 2021). The overall development method will be conducted throughout four phases, wherein ICs, nursing professionals and researchers will be involved in software requirements elicitation, prototype validation, software development, testing and operation.

For each phase, specific validation/testing initiatives will be carried out to assess the effectiveness of this proposed mHealth app. The whole DHI will be performed under an incremental and iterative (agile) approach that foresees the execution and testing of phases, as well as the necessary adjustments to any of the main deliverables for each phase.

By integrating health literacy elements, we expect that this app will also aid groups with lower levels of health literacy. The developed mHealth app may help reduce carer burden and improve the wellbeing and QoL of care recipients (Rathnayake et al., 2018).

Potential limitations of this protocol should also be noted. Technology literacy and technical issues with the mHealth tools can pose challenges to this study. To address this, we will provide a one-day technology literacy workshop on basic skills with using mobile devices and mHealth tools. By only including participants with access to mobile telephones, ‘there is a risk of social injustice by focusing on a select demographic with comparably better access to resources, sometimes referred to as the digital divide’ (Rossing et al., 2016, p. 6). However, we have attempted to address this issue by defining mobile phone access in the widest meaning feasible. Given the limitation of study resources, initially the mHealth app will only be available in Portuguese, thereby excluding participants unable to read Portuguese. Lastly, this study will not determine the effectiveness of Help2Care-PAL, as this will be the focus of a further study.

## Data Availability Statement

The raw data supporting the conclusions of this article will be made available by the authors, without undue reservation.

## Ethics Statement

The studies involving human participants were reviewed and approved by ULS-Baixo Alentejo and Polytechnic of Leiria. The patients/participants provided their written informed consent to participate in this study.

## Author Contributions

All authors listed have made a substantial, direct, and intellectual contribution to the work and approved it for publication.

## Funding

The study has received funding under the project Help2Care-PAL: Support the caregiver in palliative care, funded by Fundação “LaCaixa”. This work is also funded by national funds through FCT—Fundação para a Ciência e a Tecnologia, IP (UIDB/05704/2020 and UIDP/05704/2020) and under the Scientific Employment Stimulus—Institutional Call—[CEECINST/00051/2018].

## Conflict of Interest

The authors declare that the research was conducted in the absence of any commercial or financial relationships that could be construed as a potential conflict of interest.

## Publisher’s Note

All claims expressed in this article are solely those of the authors and do not necessarily represent those of their affiliated organizations, or those of the publisher, the editors and the reviewers. Any product that may be evaluated in this article, or claim that may be made by its manufacturer, is not guaranteed or endorsed by the publisher.
